# A rapid and accurate method of mapping invasive *Tamarix* genotypes using Sentinel-2 images

**DOI:** 10.7717/peerj.15027

**Published:** 2023-04-17

**Authors:** Solomon Wakshom Newete, Samalesu Mayonde, Thabiso Kekana, Elhadi Adam

**Affiliations:** 1 Geoinformatics Division, Agricultural Research Council—Natural Resources and Engineering, Pretoria, Gauteng, South Africa; 2 Animal Plant and Environmental Sciences, University of the Witwatersrand, Johannesburg, Gauteng, South Africa; 3 School of Geography, Archeology and Environmental Studies, University of the Witwatersrand, Johannesburg, Gauteng, South Africa; 4 Engineer Terrain Intelligence Regime, South African Army, Thaba, Tshwane, South Africa

**Keywords:** Alien invasive plants, Image classifications, Random forest, Support vector machine, Spatial distribution, Overal accuracies, Olifants river, Tamaricaceae

## Abstract

**Background:**

The management of invasive *Tamarix* genotypes depends on reliable and accurate information of their extent and distribution. This study investigated the utility of the multispectral Sentinel-2 imageries to map infestations of the invasive *Tamarix* along three riparian ecosystems in the Western Cape Province of South Africa.

**Methods:**

The Sentinel-2 image was acquired from the GloVis website (http://glovis.usgs.gov/). Random forest (RF) and support vector machine (SVM) algorithms were used to classify and estimate the spatial distribution of invasive *Tamarix* genotypes and other land-cover types in three riparian zones *viz*. the Leeu, Swart and Olifants rivers. A total of 888 reference points comprising of actual 86 GPS points and additional 802 points digitized using the Google Earth Pro free software were used to ground-truth the Sentinel-2 image classification.

**Results:**

The results showed the random forest classification produced an overall accuracy of 87.83% (with kappa value of 0.85), while SVM achieved an overall accuracy of 86.31% with kappa value of 0.83. The classification results revealed that the *Tamarix* invasion was more rampant along the Olifants River near De Rust with a spatial distribution of 913.39 and 857.74 ha based on the RF and SVM classifiers, respectively followed by the Swart River with *Tamarix* coverage of 420.06 ha and 715.46 hectares, respectively. The smallest extent of *Tamarix* invasion with only 113.52 and 74.27 hectares for SVM and RF, respectively was found in the Leeu River. Considering the overall accuracy of 85% as the lowest benchmark for a robust classification, the results obtained in this study suggests that the SVM and RF classification of the Sentinel-2 imageries were effective and suitable to map invasive *Tamarix* genotypes and discriminate them from other land-cover types.

## Introduction

Biological invasions pose serious threats to biodiversity, ecosystem functioning, animal and human health (Richardson & van Wilgen, 2004; Reynolds et al., 2020). South Africa has a long history of problems with invasive alien species, requiring urgent management interventions (Zachariades et al., 2017; van Wilgen et al., 2020). The management of invasive plant species cost South Africa an estimated ZAR 6.5 billion annually (van Wilgen & Wilson, 2018). Estimating the extent, distribution and abundance of invasive species is a prerequisite for an effective management programme, which requires identifying the hotspots to prioritize resource allocation for management (Parker et al., 2021). The continuous improvement in the spatial, spectral and temporal resolutions of remote sensing technology, has revolutionized the art of field data collection and continues to dominate the expensive and tedious practice of the traditional field survey methods. For instance, the spatial and temporal distribution of the invasive *Prosopis glanduloca* Torr. (Mesquite) in the Northern Cape Province of South Africa was accurately assessed with overall accuracy of 86% using WorldView-2 imagery (Adam, Murerirwa & Newete, 2017). The invasive aquatic macrophyte *Eichhonia crassipes* (C. Mart) Solms (water hyacinth) in Rwanda was mapped using multispectral Landsat satellite imagery with an overall accuracy of up to 87% (Mukarugwiro et al., 2019). Such accurate information supports government agencies and policy makers to prioritize resource allocation for effective and sustainable management of alien invasive species.

*Tamarix* L. (Tamaricaceae) is one of the most problematic invasive species threatening the riparian ecosystems in South Africa (Marlin et al., 2017). *Tamarix* infestations are reported to outcompete the native biota in the invaded habitats. The salt deposit in the soils directly under the canopy or the vicinity emanating from foliar salt gland-guttation and leaf littering of *Tamarix* alters the physico-chemical properties of soil by increasing salt concentration in the soil, which inhibits the growth of other co-existing seedlings, thus eventually displacing the native biodiversity (Setshedi & Newete, 2020; Araya et al., 2022). Two of the three *Tamarix* species in the South Africa are invasive, namely *Tamarix chinensis* Lour. and *T. ramosissima* Ledeb. (Marlin et al., 2017) and both are classified as category 1b invaders according to the National Environmental Management: Biodiversity Act 2014 (NEMBA) under the alien and invasive species regulations of South Africa—which compels their compulsory control. Invasive *Tamarix* species and their putative hybrids mainly occur in the humid and wet regions of the Western and Eastern Cape Provinces of South Africa (Mayonde et al., 2016; Newete, Mayonde & Byrne, 2019). Efforts to document the distribution and abundance of invasive *Tamarix* genotypes in South African riparian zones using the traditional vegetation survey method (TVSM) showed that *Tamarix* density and canopy cover were significantly greater than those of co-occurring plants (Newete, Mayonde & Byrne, 2019). The method is, however, limited to small scale surveys, and deemed not suitable and effective to determine the spatial coverage of *Tamarix* invasions at regional or national levels.

The remote sensing (RS) technique is considered as fast, and cost effective, that can provide accurate spatial and temporal information of vegetation from small scale to regional or continental scales undeterred by political boundaries or ragged terrains which bottle necks the traditional survey methods (Kumar et al., 2001; Adam, Mutanga & Rugege, 2010). Hyperspectral sensors provide high spectral resolution images, which are often expensive and more suitable for commercialization purposes because of their cost and unavailability for research. However, the low cost or freely available multispectral sensors with high spectral, temporal and spatial resolutions have proven successful in mapping vegetation at species level with 60–90% accuracy levels (Adam, Mutanga & Rugege, 2010). The mapping of *Opuntia stricta* (Haw.) Haw using Sentinel-2 imagery with an accuracy of over 80% (Muthoka et al., 2021), *Prosopis glandulosa* using WorldWiew-2 with an overall accuracy of 86% at 2 m spatial resolution (Adam, Murerirwa & Newete, 2017), and *Tamarix* species using Landsat with an accuracy of over 70% (Silván-Cárdenas & Wang, 2010) are some examples that depict the suitability of the multispectral sensors for mapping of vegetation.

The literature on *Tamarix* mapping shows different classification algorithms including, *inter alia*, the Maximum Likelihood classifier and NDVI used to map *Tamarix* invasion in Colorado River (USA) producing an overall accuracy of 80–91% (Carter et al., 2009). The Maximum Entropy (Maxent) model and associated vegetation indices were also used to discriminate *Tamarix* from co-existing species using single scene and time series analysis of Landsat 7 data achieving an overall accuracy of 90% (Evangelista et al., 2009). The spatial distribution of invasive *Tamarix* in South Africa has not yet been investigated, not at least at larger or provincial scale. Therefore, the main aim of this study was to map the extent and distribution of the invasion *Tamarix* species using Sentinel-2 satellite imagery with random forest (RF) and support vector machine (SVM), the widely used image classification algorithms.

## Materials and Methods

### Study area

**Figure 1:**
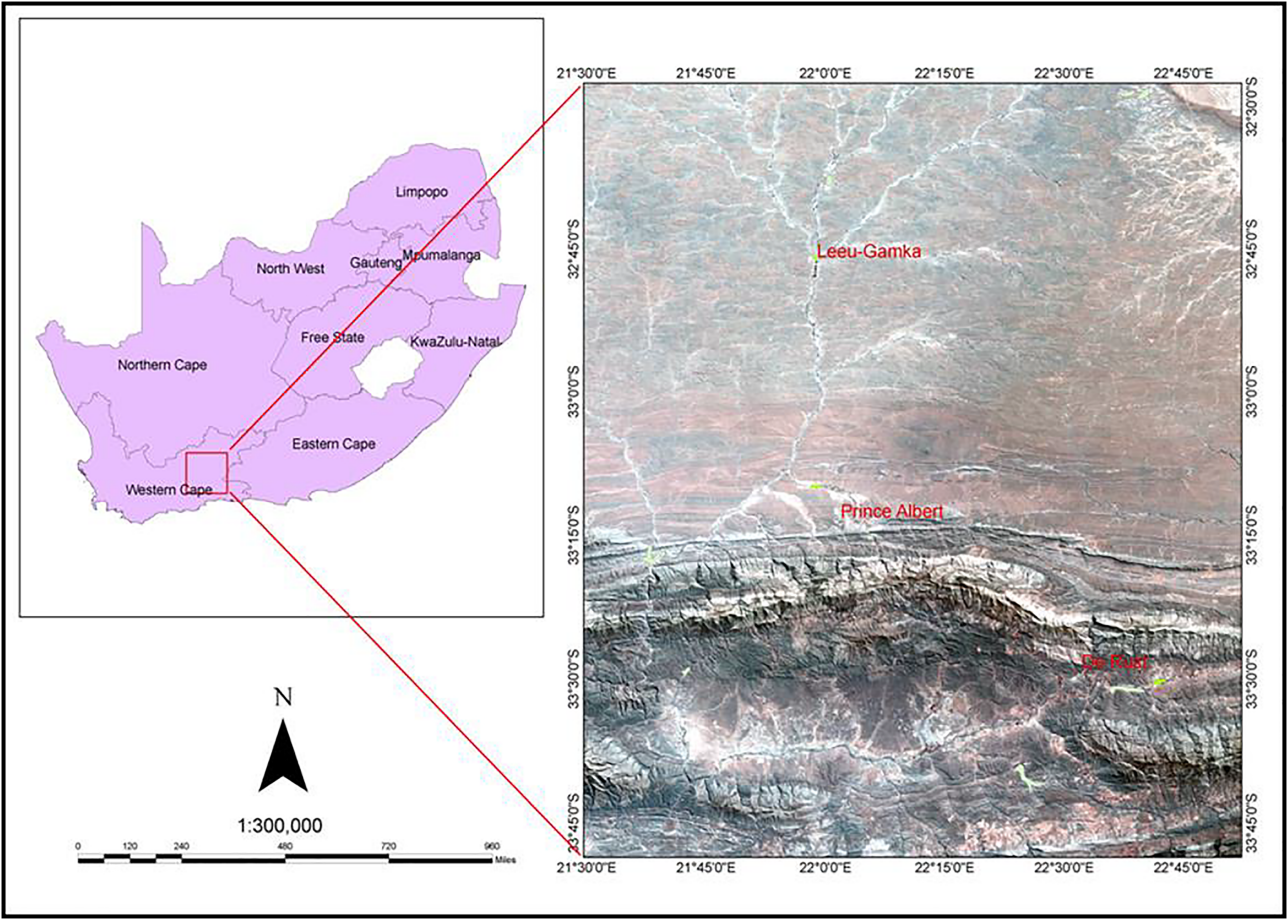
A Landsat image demonstrating the geographic location of the three study sites (Leeu-Gamka, Prince Albert and De Rust, Western Cape, South Africa).

The study was conducted along three riparian ecosystems in the Western Cape Province of South Africa. These are: the Leeu River near the Town of Leeu Gamka (32.7639°S, 21.9686°E); the Swart River (33.2167°S, 22.0333°E) situated in the Great Karoo near Prince Albert, about 72 km north of Oudtshoorn; and the Olifants River (33.4907°S, 22.5305°E) near the Town of De Rust (Fig. 1). The study areas were selected because they host the most *Tamarix* infested riparian ecosystems including the *Tamarix* hybrid genotypes (Mayonde et al., 2016; Newete, Mayonde & Byrne, 2019). They are all located in a warm and temperate climate with an average annual rainfall ranging between 50–700 mm. They have dry summer with an average temperature of 34 °C and a moist winter with low temperatures of between 3–17 °C. The sites are characterized by riparian ecosystems containing a mixture of indigenous and alien invasive plants such as *Acacia* species, *Arundo donax* L. and *Cacti* species.

### Image acquisition and pre-processing

Sentinel-2 data was used to map the distribution of the invasive *Tamarix* genotypes and other land-use land cover types. Sentinel-2 multispectral images are characterized by high spatial and spectral resolutions with 13 spectral bands in the visible, near-infrared and shortwave infrared regions. They have spatial resolution of 10 m for bands 2, 3, 4 and 8; and a 20 m spatial resolution for bands 5, 6, 7, 8A, 11 and 12 (Adagbasa, Adelabu & Okello, 2019). The three wavebands located in the red edge section of the electromagnetic spectrum (EMS) centered at 705, 740 and 783 nm are critical for detecting vegetation characteristics such as chlorophyll, leaf area index and general species identification—all of which are used to identify and map vegetation using Sentinel-2 imageries (Pu et al., 2003; Adagbasa, Adelabu & Okello, 2019; Masemola, Cho & Ramoelo, 2020). The Sentinel-2 image was acquired on a sunny and clear day of the 9^th^ of November 2018 from the GloVis web-link (http://glovis.usgs.gov/). Copernicus Sentinel-2 mission is an optical earth observation mission with two satellites constellation obits around the Earth at an altitude of 786 km (https://sentinel.esa.int). The sensor has a 290 km swath width, a 5-day temporal resolution, and 13 spectral bands at 10, 20 and 60 m spatial resolutions. Sentinel-2 images were atmospherically corrected using Sen2cor plugin in SNAP 6.0.5 software by performing atmospheric, terrain and citrus correction. This processor created surface reflectance image and terrain corrected images.

### Reference data and image classification

Reference data points for ground-truthing of the Sentinel-2 image were collected in November 2017, during the flowering stage of *Tamarix* to enhance discrimination from other co-existing plants species. Field data collection was conducted close to the time the Sentinel-2 multi-spectral image used in this study was acquired. A total of 888 reference data points were used in this study comprising of 86 coordinates collected from *Tamarix* and other woody vegetation such as *Vachellia karroo*, among others using the handheld Global Positioning System (GPS) receiver (Garmin eTrex). The additional 802 reference points for other land use and land cover (LULC) types (*i.e*., Agriculture, Buildup, Waterbody and bare land and Low dense grassland) were obtained by visual interpretation using the Google Earth Pro software. The two widely used classification algorithms for mapping vegetation, random forest (RF) and support vector machine (SVM), were used to classify the Sentinel-2 satellite image. Random forest is a classification model developed based on bagging operation where a number of multiple classification trees are derived from a random subset of training data to improve classification accuracy. The RF classifier deal with outliers and noise using the two parameters, *mtry*, which is the number of variables to split nodes individual trees, and *ntree*, the number of trees to be grown *(Breiman, 2001*). Similar to RF, SVM is a non-parametric algorithm that seeks an optimal discrimination hyperplane by minimizing the upper bound of the discrimination error (Xu et al., 2020). The advantage of using SVM is the ability to discriminate between species using optimization algorithms. SVM is sensitive to noise or overtraining and it can deal with unbalanced data as well as small sample sizes (Tehrany, Kumar & Shabani, 2019). The reference data was randomly split into training (70%) and validation (30%) data to train both classifiers and validate the accuracy of the Sentinel-2 image classifications (Table 1). Validation data was used to construct a confusion matrix table.

**Table 1:** Reference data for the different land-cover classes, *Tamarix* and other vegetation for training and validation in mapping invasive *Tamarix* in South Africa using Sentinel-2 image.

Land-cover type	Codes	Training data	Test data	Total
Agriculture	A	96	40	136
Bareland	BL	115	49	164
Buildup	B	89	37	126
*Tamarix*	T	148	63	211
Woody vegetation	WV	114	48	162
Water body	W	63	26	89

### Accuracy assessment

An accuracy assessment was performed to establish the reliability and validity of the classified maps developed using RF and SVM classifiers. The confusion matrix table showing agreement between the classification result and reference imagery (ground pixels) was used to compute the overall, producer’s, and user’s accuracies as well as the kappa coefficient *(de Leeuw et al., 2006*). User accuracy (UA) describes the amount of error omission by the user and shows the probability that a pixel labelled as specific *Tamarix* and land cover types in the map is the actual class. Producer’s accuracy (PA) describes the amount of errors of commission and shows that the specific *Tamarix* and land cover types on the ground are accurately classified. Overall accuracy (OA) refers to the percentage probability that the random selected pixel is correctly classified in land use and land cover thematic map. The kappa coefficient was used to remove the chance agreement during image classification. Mcnemar test was employed to test the performance difference of SVM and RF. At a significance level of 5% the difference in accuracy is considered to be statistically significant when the Z value is greater than 1.96.

## Results

### Random forest and support vector machine tuning

**Figure 2:**
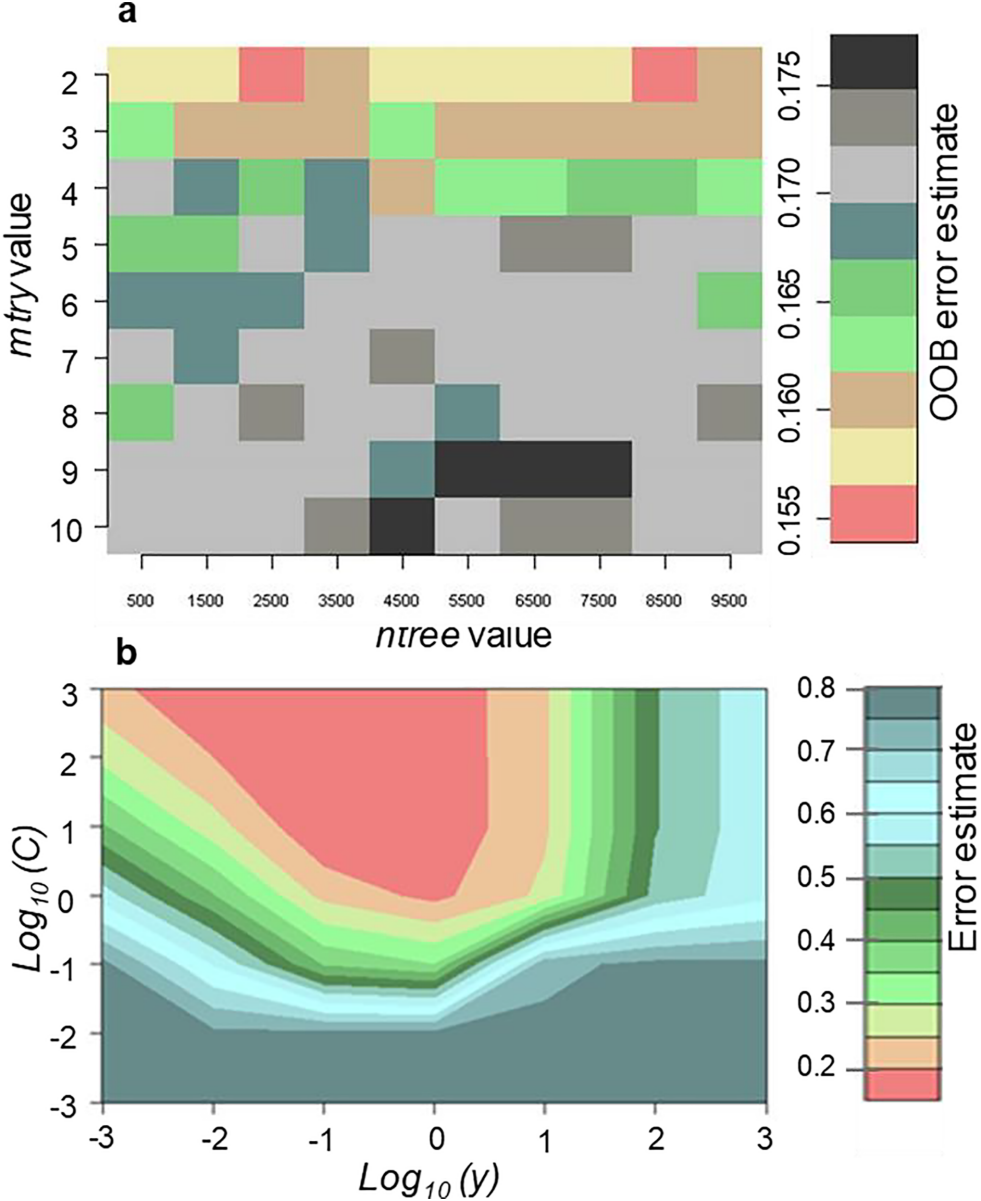
Tuning of (A) RF parameters (*mtry* and *ntree*) with the OOB sample used to determine the error and (B) SVM parameters (γ and C) after optimization utilizing the 10-fold cross validation sampling method.

Random forest was optimised to accurately determine the best input parameters for classification of invasive *Tamarix* and five land-cover classes using 10 cross validations. Results showed that merger of *mtry* value of 2 and *ntree* value of 8,500 produced the lowest Out-of-Bag (OOB) error of 15.51%. On the other hand, the highest OOB error produced was 17.5% which resulted from the combination of *mtry* value of 10 and *ntree* value of 4,500 (Fig. 2A). Thus, the combination of *mtry* value of 2 and *ntree* value of 8,500 was selected and used. For the SVM tuning, the radial kernel method using the *gamma* and *cost* parameters were optimised to accurately determine the best combination with the lowest error. The combination of *gamma* and *cost* values of 0.1 and 10, respectively produced the lowest classification error of 15.35% (Fig. 2B).

### The performance of RF and SVM in *Tamarix* classification

**Figure 3:**
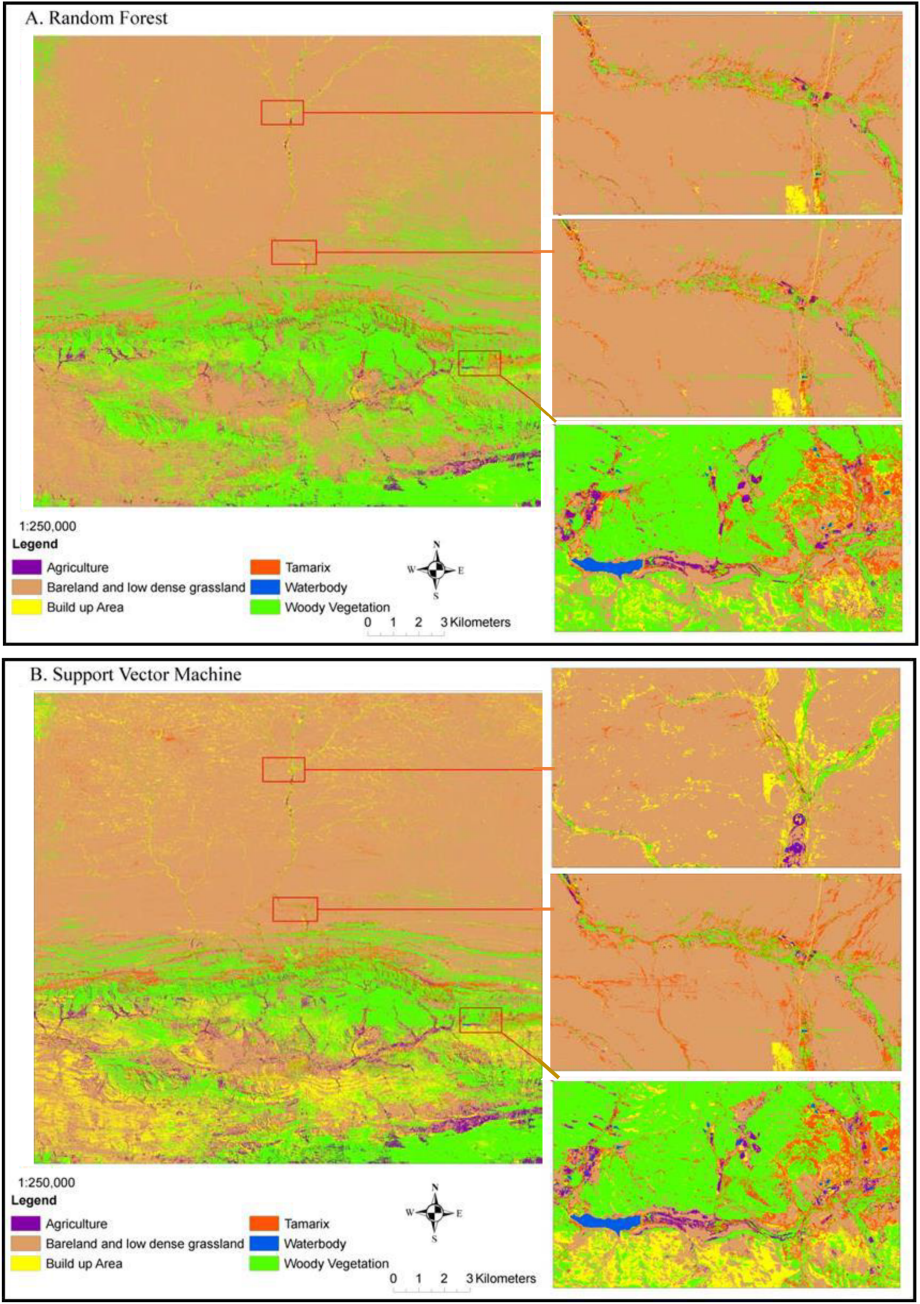
Mapping current *Tamarix* distribution and other land-cover types in the Western Cape Province using Sentinel-2 data, (A) random forest classifier and (B) support vector machine classifier.

The RF and SVM algorithms classified the alien invasive *Tamarix* species with significant accuracy level (Fig. 3). The dominant land-cover classified was bare land followed by low-dense grassland and Woody vegetation. *Tamarix* was dominant in low lying areas along the banks and flood plains, while other woody plants, predominantly the *Vachallia karroo*, were found on both low lying and high mountainous areas (Fig. 3). Our results showed a clear ecotone between *Tamarix*, other woody plants, bare land and low-dense grassland. Random forest classified *Tamarix* trees, woody plants and Agriculture land-cover (Fig. 3A) much clearer than the SVM classifier (Fig. 3B). However, the Built up areas were more notable in the SVM classified map (Fig. 3B) than in the RF classifier (Fig. 3A).

**Figure 4:**
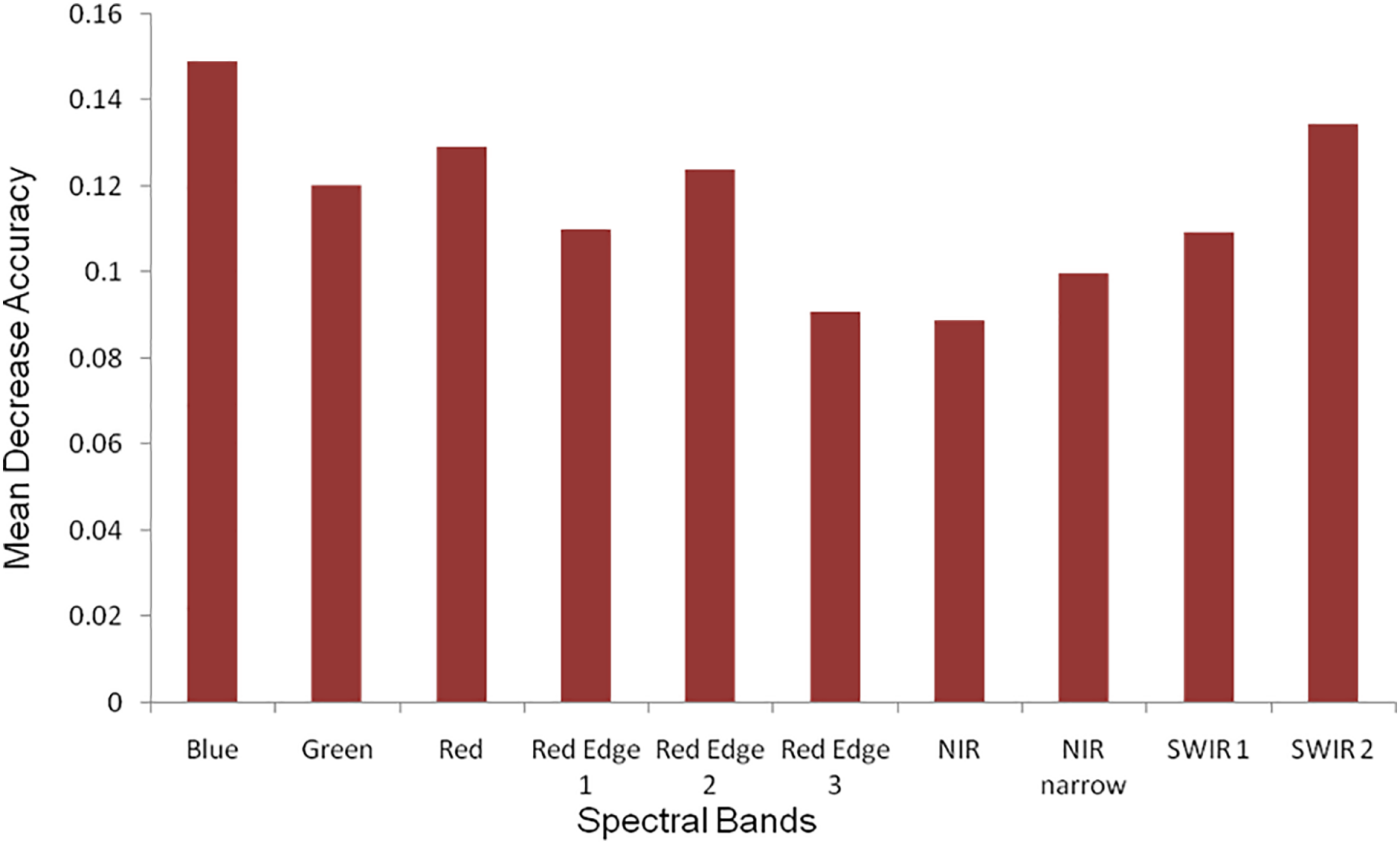
The measure of the Sentinel-2 (10 m spatial resolution) bands variable importance in RF classification process.

During the classification process RF provided variable importance indicating the character of each band in image classification. The most significant bands were red, blue, green, SWIR 2 and red edge with the highest mean decrease in accuracy (Fig. 4).

**Figure 5:**
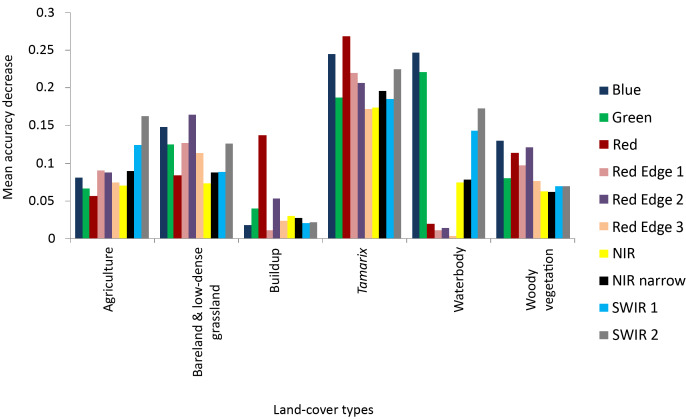
The relationship between invasive *Tamarix* spp, other Woody vegetation and land cover class, and the RF variable importance.

Red, blue, red edge 1, red edge 2 and SWIR 2 bands were found to be the most important bands in *Tamarix* classification and other vegetated land-cover types, while SWIR 2, green and blue bands were important bands in classification of water bodies (Fig. 5). Near infrared short wave, red edge 1, red edge 2 and green bands were important in the classification of bare land and low-dense grassland, while the useful bands in classification of Built areas were red edge 2 and red edge 3 (Fig. 5).

### Accuracy assessment

Random forest produced a higher OA of 87.83% with a kappa value of 0.85 compared to SVM (86.50% and kappa of 0.83) (Tables 2 and 3). However, a spectral confusion was found between bare land and low-dense grassland, *Tamarix* and build-up area, resulting in a lower PA of 67.57% in classifying the build-up cluster. In contrast, the classification of the water bodies showed no spectral confusion producing both user’s and producer’s accuracies of 100%. The other spectral confusion was observed between bare land and low-dense grassland, Agriculture and build-up area when classifying bare land and low-dense grassland producing low UA of 80.77%.

**Table 2:** Confusion matrix for validating 2018 land use and land cover (LULC) map. The RF accuracies: Overall (OA), Producer (PA) and User (UA) were determined from Sentinel 2 image.

Class	A	BL	B	T	W	WV	Row total	UA
Agriculture (A)	33	1	1	2	0	0	37	89.19
Bareland (BL)	4	42	6	0	0	0	52	80.77
Buildup (B)	1	2	25	0	0	1	29	86.21
*Tamarix* (T)	0	2	3	61	0	3	69	88.41
Waterbody (W)	0	0	0	0	26	0	26	100
Woody vegetation (WV)	2	2	2	0	0	44	50	88
Column total	40	49	37	63	26	48	263	
PA	82.5	85.71	67.57	96.83	100	91.67		

**Note:**

Overall accuracy (OA) = 85.83% and Kappa = 0.851. NB: Note that A, Agriculture; BL, Bareland and low dense grassland; B, Build-up area; T, *Tamarix*; W, Waterbody; WV, Woody Vegetation.

**Table 3:** Confusion matrix for validating 2018 classified Sentinel 2 image. SVM accuracies User (UA), Overall (OA), and Producer (PA).

Class	A	BL	B	T	W	WV	Row total	UA
Agriculture (A)	35	2	2	0	0	2	41	85.57
Bareland (BL)	3	40	2	0	0	0	45	88.89
Buildup (B)	1	3	28	2	0	0	34	82.35
Tamarix (T)	0	2	3	60	0	8	73	81.2
Waterbody (W)	1	0	0	0	26	0	27	96.3
Woody vegetation (WV)	0	2	2	1	0	38	43	88.37
Total (Column)	40	49	37	63	26	48	263	
PA	87.5	81.63	75.68	95.24	100	79.17		

**Note:**

Overall accuracy (OA) = 86.31% and Kappa = 0.833. Note that A, Agriculture; BL, Bareland and low dense grassland; B, Build-up area; T, *Tamarix*; W, Waterbody; WV, Woody Vegetation.

The confusion matrix of SVM produced an Overall Accuracy (OA) of 86.31% and Kappa value of 0.833 (Table 3). Spectral confusion was noted between Build-up areas, woody plants and Agricultural lands. Thus, low producer’s and user’s accuracies of 75.68% and 82.35% were reported, respectively. Confusion was also observed between *Tamarix* and other woody vegetation leading to a user’s accuracy of 81.2% (Table 3).

Although both RF and SVM had different classification accuracies, Mcnemar test showed that there was no significant difference at 5% significant level as the Z value was 0.75 which was less than 1.96.

### Area cover of the invasive *Tamarix* and other land-cover types

The area (ha) of all the six land-cover types were successfully estimated for all three study sites for both RF and SVM classifiers of the Sentinel-2 data (Table 4). The Bareland was the dominant land cover in Leeu-Gamka and Prince Albert for both RF and SVM, while the two classifiers showed the Woody vegetation to be the most dominant land-cover in De Rust (Table 4). Agricultural lands and water bodies were found to cover the smallest areas in all three study sites for both RF and SVM. The invasive *Tamarix* was found to cover a bigger area in De Rust (Olifants River) compared to other study sites with RF having a greater classification percentage (16.04%). However, the SVM showed almost double the area coverage with 6.7% *Tamarix* cover compared to the 3.9% of RF in Prince Albert (Swart River). A similar trend was observed in Leeu-Gamka (Leeu River) where SVM showed a larger area cover for *Tamarix* plants than RF. Leeu-Gamka had the smallest land cover of invasive *Tamarix* compared to other sites with 3.52% and 2.12% area cover identified by SVM and RF respectively. In general, the classification of all land-cover types were similar for SVM and RF in all three study sites with a low difference of 0.5% in Leeu-Gamka, 1.04% in Prince Albert and a 0.09% in De Rust in the performance of the two classification algorithms (Table 4).

**Table 4:** Area estimates in hectare (ha) and percentage (%) for six land-cover classes in three study sites obtained by SVM and RF classifiers for mapping of invasive *Tamarix* using Sentinel-2 satellite image.

Study site	Class type	SVM area (ha)	SVM (%)	RF area (ha)	RF (%)	+/− (%)
Leeu River—Leeu Gamka	Agriculture	50.1	1.43%	69.07	1.97%	−0.54%
	Bareland	2,778.73	79.44%	2,920.53	83.49%	−4.05%
	Buildup area	384.23	10.98%	259.89	7.43%	+3.55%
	*Tamarix*	113.52	3.25%	74.27	2.12%	+1.12%
	Waterbody	3.3	0.09%	0.41	0.01%	+0.08%
	Woody vegetation	168	4.80%	173.71	4.97%	−0.16%
Swart River—Prince Albert	Agriculture	96.48	0.90%	86.77	0.81%	+0.09%
	Bareland	9,332.03	86.92%	9,515.69	88.63%	−1.71%
	Buildup area	240.58	2.24%	276.2	2.57%	−0.33%
	*Tamarix*	715.46	6.66%	420.06	3.91%	+2.75%
	Waterbody	6.77	0.06%	2.29	0.02%	+0.04%
	Woody vegetation	344.56	3.21%	434.87	4.05%	−0.84%
Olifants River—De Rust	Agriculture	308.34	5.42%	288.56	5.07%	+0.35%
	Bareland	1,507.95	26.48%	1,488.4	26.14%	+0.34%
	Buildup area	539.94	9.48%	461.29	8.10%	+1.38%
	*Tamarix*	857.74	15.06%	913.39	16.04%	−0.98%
	Waterbody	134.67	2.37%	123.5	2.17%	+0.20%
	Woody vegetation	2,345.51	41.19%	2,419.01	42.48%	−1.29%

## Discussion

The utility of Sentinel-2 multispectral imagery to distinguish *Tamarix* genotypes from other land cover types and determine the spatial distribution of the invasive species along riparian ecosystems in the Western Cape Province of South Africa was tested. Although ground surveys are still commonly used to determine the extent and distribution of alien invasive plants and other vegetation, mapping with remote sensing has increasingly become important for researchers and natural resource managers (Parker et al., 2021). Alien invasive species are widely recognized as the second largest threat to biodiversity after direct habitat destruction. They outcompete native biota for natural resources, thus reducing indigenous species abundance and richness, which subsequently impairs ecosystem functioning (Pyšek et al., 2012; Hejda, Pyšek & Jarošík, 2009). Remote sensing can accurately determine the present and past distribution of invaders to better assess their environmental impacts, forecast potential spreads, and develop effective control strategies (Medlin et al., 2000; Adam, Mutanga & Rugege, 2010; Parker et al., 2021).

The availability of high-resolution satellite data provides a great opportunity to map and monitor the distribution of alien invasive *Tamarix*. These plants cause serious negative impacts on the most vulnerable riparian ecosystems and the biodiversity in South Africa (Setshedi & Newete, 2020; Araya et al., 2022). Nevertheless, no study has shown the spatial distribution of this notorious invader before. Newete, Mayonde & Byrne (2019) attempted to quantify the distribution of the invasive *Tamarix* species using the traditional survey method and showed that *Tamarix* dominated the riparian ecosystems displacing many co-occurring indigenous plants in the Eastern and Western Cape provinces of the country. The relatively high accuracies achieved using RF (87.83%) and SVM (86.31%) in the current study demonstrated that the high-resolution Sentinel-2 data is capable of discriminating *Tamarix* from other vegetation land covers at 10 m spatial resolution. Our results are similar to the overall accuracy (OA) of 85.07% obtained to discriminate invasive Eucalyptus from other land covers using Sentinel-2 data by Sibanda et al. (2021). The OA obtained in this study is also relatively higher than the 77% obtained when Sentinel-2 data was used to map the invasive *Baccharis halimifolia* in Spain (Calleja et al., 2019). However, relatively higher OA of 94.4% for both RF and SVM classifiers was obtained to map invasive sea grass in the Mediterranean Sea in Greece using Sentinel-2 data (Traganos & Reinartz, 2018). Similarly, relatively higher overall accuracies of 80–90% was reported using a hyperspectral data (Hamada et al., 2007) and a high resolution multispectral data such as Quickbird (Ji & Wang, 2015) to map *Tamarix* species. Strangely, this study obtained a low Producer’s Accuracy (PA) of 67.57% (RF) and 75.68 (SVM) for the Build-up land cover, which is the lowest for all the analysed land cover types. Although Build-up areas are reasonably easy to classify, both algorithms showed some spectral confusions with Bareland and Low dense grass land cover type. The level of classification accuracy found in the current study was, therefore, comparable, and relevant for accurate classification of the invasive *Tamarix* genotypes. As an alternative monitoring tool, Sentinel-2 data has the potential to resolve some of the unique properties of the high resolution hyperspectral data, which is often limited by its availability due to its enormous cost as well as its data dimensionality and redundancy (Adam, Mutanga & Rugege, 2010; Cho, Malahlela & Ramoelo, 2015).

Detecting a specific plant species in natural landscapes such as rangelands and riparian ecosystems using remote sensing could sometimes be challenging because of the high level of heterogeneity in the vegetation composition (Anderson et al., 1993; Lass et al., 2005). However, large-scale infestations where the invasive species is the dominant plant is relatively easier to detect remotely (Evangelista et al., 2009). When characterizing the invasive *Tamarix* in the Western Cape, the most optimal Sentinal-2 spectral variables using the RF algorithm were provided by red, blue, green, SWIR 2 and red edge. These bands are associated with plants specific characteristics such as chlorophyll, leaf area index and canopy cover (Adelabu, Mutanga & Adam, 2014). Although, RF was 1% greater than the SVM classifier, the difference between the two algorithms was not significant. The advantage of random forest is that it can provide variable importance, which determines the importance of each band in the Sentinel-2 image (see Fig. 4) (Breiman, 2001). However, different kernels can be used in SVM classification with radial kernel used in the study because it is an efficient method to deal with vegetation species class inseparability issues (Adam, Murerirwa & Newete, 2017; Sabat-Tomala, Raczko & Zagajewski, 2020). Our high accuracy levels obtained for both RF and SVM classifiers with an insignificant difference between them correspond with other studies where RF and SVM were applied on multispectral data (Adam et al., 2012; Traganos & Reinartz, 2018; Adam, Murerirwa & Newete, 2017; Abutaleb et al., 2021) and hyperspectral (Hamada et al., 2007; Sabat-Tomala, Raczko & Zagajewski, 2020) data for vegetation mapping. Therefore, both RF and SVM algorithms can be effectively used to map alien invasive *Tamarix*.

Our study is the first to quantify the spatial coverage of invasive *Tamarix* in the Western Cape province of South Africa. The RF and SVM classifiers showed the current distribution of invasive *Tamarix* which covers between 2.12–3.25% (74.27–113.52 ha) of the land in Leeu Gamka and 3.91–6.66% (420.06–715.46 ha respectively) in Prince Albert. However, a large variation obtained between RF and SVM in estimating *Tamarix* distribution in Swart River in Prince Albert could be due to misclassification. For example, eight bands spectral confusion between *Tamarix* and woody vegetation was obtained by SVM algorithm while RF resulted in three spectral bands confusion between the two classes, suggesting that SVM over-estimated *Tamarix* distribution. Interestingly, our results showed 15.06–16.04% (923.39–857.74 ha respectively) of the riparian zones along the Olifants River in De Rust is covered by the alien invasive *Tamarix* trees, indicating that the site has the largest *Tamarix* invasion compared to the Leeu River in Leeu Gamka and Swart River in Prince Albert. Amongst many factors, hybridization is believed to augment *Tamarix* invasion, hybrids have been recorded to be the dominant invading genotypes in South Africa and the United States (Gaskin & Kazmer, 2009; Mayonde et al., 2016). The spatial distribution of the *Tamarix* genotypes reported in this study also affirms the *Tamarix* molecular analysis reported by Newete, Mayonde & Byrne (2019), where they showed the Olifants River to be dominated with *T. chinensis* × *T. ramosissima* hybrids. The low land cover by the invasive *Tamarix* in Leeu-Gamka and Prince Albert could be due to the presence of the indigenous *T. usneoides* (Mayonde et al., 2015; Mayonde et al., 2019), resulting in a heterogenous composition of indigenous and invasive *Tamarix* species. Despite the advantages of Sentinel-2 in vegetation classification, this satellite imagery can still hinder individual tree-level mapping of alien invasive species in highly heterogenous landscape due to spectral confusion. Especially when the Sentinel-2 data was acquired from a single phenological period of the vegetation, like in our study, which can result in unsatisfactory accuracy levels for single species detection. Therefore, we recommend the use of time series analysis because it can provide continuous information of the species, and the data can be integration in the discriminating algorithm leading to increased detection accuracy (Müllerová et al., 2017; Masemola, Cho & Ramoelo, 2020).

Our study showed that RF outperformed SVM in classifying the distribution of the invasive *Tamarix* in De Rust as opposed to those observed in Leeu-Gamka and Prince Albert. The presence of the morphologically similar *T. chinensis* and *T. ramosissima* in De Rust could favour RF because each classifier depends on the values of the independently sampled random vectors (Breiman, 2001). The RF classifier algorithms are more robust to noise and outliers because the random vectors have the same distribution power for all classifiers in the forest, giving equal power for each tree to contribute a unit to the overall input of a particular class (Breiman, 2001). However, SVM is said to be a versatile classifier algorithm that forms models using small data instances (support vectors) from different classes (Mountrakis, Im & Ogole, 2011; Abedi, Norouzi & Bahroudi, 2012)—hence, it could easily group together the indigenous *T. usneoides* with the alien invasive *T. chinensis* and *T. ramosissima*. Biological control management programmes in South Africa, will target *Tamarix* infested areas (Byrne et al., 2021) should the current trial for *Tamarix* biocontrol agents in the country proves effective and safe, which will require accurate estimate of the extent and distribution of the invasive *Tamarix* species to identify hotspots for release. Over the years, remote sensing has earned enormous popularity as a suitable tool for mapping and monitoring invasive species providing information about plant invasion dynamism (Parker et al., 2021). Although the data was collected in 2017 which could be regarded as the limitation of this study, we recommend an updated analysis of the recent spatial distribution of the invasive *Tamarix* genotypes. However, our assessment recommends allocation of resources for chemical or mechanical control of invasive *Tamarix* genotypes along the riparian areas of the Olifants River in De Rust where it is pervasive, until an effective biocontrol agent is approved for release against in South Africa.

## Conclusions

The study investigated the extent and distribution of the alien invasive *Tamarix* genotypes using the multispectral Sentinel-2 imagery, with random forest (RF) and support vector machine (SVM) algorithms. The results showed that the alien invasive *Tamarix* was more prevalent in the Olifants River in De Rust with a spatial coverage of 913.39 ha (RF) and 857.74 (SVM) representing 16.04% and 15.06% of the study area, respectively compared to other land use types. The smallest spatial coverage was reported in the Leeu River with 74.27 and 113.52 ha detected for RF and SVM, respectively. Sentinel-2 data proves effective in mapping *Tamarix* genotypes accurately.

## Supplemental Information

10.7717/peerj.15027/supp-1 Supplemental Information 1 The raw data showing the waypoints (Global Positioning System—GPS coordinates) of the land use land cover types for ground truthing.

## Additional Information and Declarations

### Data Availability

The following information was supplied regarding data availability: The raw data is available as a Supplemental File. The satellite images are available at Figshare: Mayonde, Samalesu (2022): Raw data of Sentinel-2 satellite images used to map invasive Tamarix in South Africa. figshare. Dataset. https://doi.org/10.6084/m9.figshare.20509650.v1.
